# Prevalence of *pediculosis capitis* and associated factors among schoolchildren in Woreta town, northwest Ethiopia

**DOI:** 10.1186/s13104-019-4521-8

**Published:** 2019-07-30

**Authors:** Henok Dagne, Awel Aba Biya, Amanuel Tirfie, Walelegn Worku Yallew, Baye Dagnew

**Affiliations:** 1 Department of Environmental and Occupational Health and Safety, Institute of Public Health, University of Gondar (UoG), P.O. Box 196, Gondar, Ethiopia; 2 Department of Human Physiology, School of Medicine, UoG, P.O. Box 196, Gondar, Ethiopia

**Keywords:** *Pediculosis capitis*, Schoolchildren, Ethiopia

## Abstract

**Objectives:**

The aim of the study was to determine the associated risk factors and prevalence of pediculosis capitis among school-aged children in Woreta town, northwest Ethiopia. An institution-based cross-sectional study was carried out on 402 schoolchildren in Woreta town public schools from grades 1 to 4 students conducted from April to June 2018. After selection by simple random sampling, face to face interview and observations were performed using a semi-structured pre-tested questionnaire. Data were entered into EPI Info 7 and exported to SPSS 21 for further analysis. Descriptive results were presented by simple frequency, percentage, and mean. Binary logistic regression was used to identify associated factors. Those variables with a p-value ≤ 0.05 in the multivariable logistic regression were declared as significantly associated with *pediculosis capitis* infestation.

**Result:**

The prevalence of *pediculosis capitis* was 65.7% [95% CI 60.01–70.3%]. Sex of child, age of the child, maternal education, sharing hair comb, knowledge, and attitude towards *pediculosis capitis* infestation and hygiene practice were significantly associated with pediculosis (a p-value ≤ 0.05). Pediculosis infestation is found to be a major public health problem which demands special attention of the community and the government at large particularly the health sector to reduce the problem.

## Introduction

Lice are human-specific ectoparasites and blood-sucking insects which are known to cause trench fever, epidemic typhus, and relapsing fever [1–3]. It affects all strata of the society infesting the hair and skin of humans as *pediculus capitis* (head lice), *Pediculus humanus* and *Phthiris pubis* [4]. Infestation by lice is a major public health problem globally. It is most common in resource-limited countries [5]. It is a ubiquitous problem in children [6]. *Pediculosis capitis* can cause sleep loss, irritation, pruritus, discomfort, secondary bacterial infections (such as impetigo and acute glomerulonephritis), and lymphadenopathy [7, 8]. Head lice infestations can occasionally cause mental disorders [9]. Several studies from various regions in the world have reported that *pediculosis capitis* infestation prevalence varies from country to country. Studies conducted in southeast Iran reported 67.3% [10], Bangkok, Thailand 23.32% [11], Bilbao, Spain 9.39% [12] prevalence of *pediculosis capitis* infestation among schoolchildren. It is recommended that local research to obtain evidence on epidemiology, knowledge, and attitudes on lice infestation to find effective medications as currently there are no available data on the prevalence of pediculosis capitis in Woreta town [13]. Although pediculosis infestation is a major public health problem, there is lack of evidence in the study area. The study will help authorities to allocate resources and target risk factors to reduce the burden of infestation. Therefore, the current study aimed to assess the prevalence and associated factors of infestation by *Pediculus humanus capitis* among schoolchildren in Woreta town, 2018.

## Main text

### Methods

#### Study setting and period

The study was conducted on schoolchildren in Woreta town public first cycle (grades 1 to 4) elementary schools from April to June 2018. The town is located at 2092 meters above sea level, 589 km far from Addis Ababa, the capital city of Ethiopia. There were three elementary schools namely Woreta (formerly known as Guaya), Dudemegn and Esteber with a total number of students of 1419, 1320 and 500 respectively.

##### Study design

Institution-based cross-sectional study was employed.

#### Study population and eligibility criteria

Schoolchildren from grades 1 to 4 who were available during the period of data collection whose guardians/parents have given assent and who agreed to participate, and who did not have active head scabies were included.

#### Sample size determination and sampling technique

The sample size was determined by using a single population proportion formula [14] with assumptions; p = 50% (as there was no previous study in the country), 95% confidence level ($Z_{a/2}$) and margin of error (d) = 0.05

$$n = \frac{{\left( {z_{{\alpha /2}} } \right)^{2} p(1 - p)}}{{d^{2} }} = \frac{{\left( {1.96} \right)^{2} 0.5(1 - 0.5)}}{{0.05^{2} }} = 384.$$

Considering 5% of the non-response rate, the total sample size was 402. Samples were selected by simple random sampling technique and allocated proportionally to the three schools based on the number of students at each school.

#### Operational definitions

##### Pediculosis

A child with at least one head louse by wet combing is considered as being infested by *pediculosis capitis* [15].

##### Schoolchildren

Children attending classes from grades 1 to 4 were regarded as schoolchildren in the current study.

##### Knowledge

Knowledge was assessed by 10 knowledge items with yes/no category. Students who scored mean and above mean of knowledge questions were considered as knowledgeable.

##### Attitude

Attitude was measured by 8 attitude questions with a 5-scale Likert (1-strongly disagree to 5- strongly agree). Children who scored mean and above of the attitude questions were considered as having a good attitude.

##### Practice

Children were asked five practice questions regarding *pediculosis capitis* prevention behavior. Those children who scored mean and above mean of the practice questions were considered as having a good practice.

#### Data collection instrument, procedure and quality control

A pre-tested, semi-structured questionnaire containing socio-demographic variables, behavioral characteristics (knowledge, attitude and practice regarding *pediculosis capitis* infestation) was used. Wet combing and observation was used to decide whether a child is infested or not according to Wegner [15]. Interview and observation was done by two Environmental health bachelor degree students after receiving training about the data collection tool, techniques, the purpose of the study and ethical issues. The training was given by the principal investigator at University of Gondar, College of Medicine and Health Science.

##### Reliability

The Cronbach’s alpha scale for knowledge, attitude and practice questions were 0.84 (good), 0.97 (excellent) and 0.70 (acceptable) reliability, respectively. The overall Cronbach’s alpha result was 0.77 (acceptable) [16].

##### Validity

Content validity was ensured by pretesting the data collection tool on 20 students out of the study area. The tool was modified based on the observed findings from the pre-test result. Some questions were rewritten for better understanding of study participants. Words having ambiguous meaning were corrected.

#### Data processing and analysis

Data were entered into EPI Info 7 and exported to SPSS 21 for analysis. Mean, frequency and percentage were used for description. A binary logistic regression model was used to identify significantly associated variables at a p-value ≤ 0.05. During bivariable analysis, variables with a p-value ≤ 0.2 were candidates for multivariable logistic regression for the final model. Hosmer and Lemeshow goodness-of-fit test was performed.

### Result

#### Socio-demographic characteristics

Four hundred two schoolchildren with a mean age of 10.19 ± 1.62 years participated in this study. About 186 (46.3%) were males (Table 1). Two hundred and thirty-eight (59.2%) students reported taking bath once per week, 296 (73.6%) sleep with others, and 187 (46.5%) share comb (Table 2).

**Table 1:** Sociodemographic characteristics of study participants school Woreta town, 2018 (n = 402)

Variables	Categories	Frequency	Percent (%)
Student grade level	Grade 1	99	24.6
	Grade 2	99	24.6
	Grade 3	100	24.9
	Grade 4	104	25.9
Sex	Male	186	46.3
	Female	216	53.7
Age	5–8	85	21.1
	9–11	230	57.2
	> 12	87	21.6
Religion	Orthodox	291	72.4
	Muslim	108	26.9
	Protestant	3	0.7
Fathers education	Illiterate	111	27.6
	Elementary	161	40
	Secondary and above	130	32.3
Mothers education	Illiterate	171	42.5
	Elementary	162	40.3
	Secondary and above	69	17.2
Fathers occupation	Private worker	153	38.1
	Government worker	64	15.9
	Daily labor	42	10.4
	Others	143	35.6
Mother’s occupation	Private worker	62	15.4
	Government worker	25	6.2
	Housewife	240	59.7
	Others	75	18.7
Family size	2 to 3	21	5.2
	4 to 5	206	51.2
	6 to 7	136	33.8
	> 8	39	9.7

**Table 2:** Behavioral characteristics of respondents, Woreta town (n = 402)

Variable	Category	Frequency	Percent (%)
Sleeping arrangement	Alone	106	26.4
	With others	296	73.6
Sharing of hair comb	Yes	187	46.5
	No	215	53.5
Frequency of combing hair	Once a week	166	41.3
	Twice a week	100	24.9
	Thrice a week	74	18.4
	Never	62	15.4
The class situation for learning	Comfortable	46	11.4
	Not comfortable	356	88.6
Presence of hygiene inspection club	Yes	106	26.4
	No	296	73.6
Frequency of class inspection	Once week	107	26.6
	Twice a week	1	0.2
	Never	294	73.1
Water accessibility in the school	Yes	29	7.2
	No	373	92.8

Two hundred and sixty-four (65.7%) [95% CI 61.01–70.3%] students were infested by *pediculosis capitis*.

#### Factors associated with *Pediculosis Capitis* infestation

In bivariable analysis; grade level, sex and age of children, maternal education and occupation, family size, sleeping arrangement, sharing of hair comb, knowledge, attitude and practice towards pediculosis were candidate variables (p-value ≤ 0.2) for multivariable logistic regression. Only age of child, sex of a child, maternal education, sharing hair comb, knowledge, attitude and practice were significantly associated with *pediculosis capitis* infestation.

Females were 3.29 times [AOR = 3.29, 95% CI (1.94, 5.59)] more infested by *pediculosis capitis* than males. The odds of *pediculosis capitis* infestation was twice among students aged 9 to 11 years than aged above 12 years [AOR = 2.04, 95% CI (1.07. 3.87)].

Students having illiterate mothers were 3.57 [AOR = 3.57, 95% CI (1.74, 7.33)] times at risk of being infested than those with mother’s education greater than elementary level.

Students who shared hair comb were 2.72 [AOR = 2.72, 95% CI (1.58, 4.69)] times being infested than those who did not share hair comb. Those with poor knowledge were 2.51 [AOR = 2.51, 95% CI (1.24, 5.10)] times, students with a poor attitude were 2.42 [AOR = 2.42, 95% CI (1.28, 4.60)] times and children with poor practice were 3.84 (AOR = 3.84, 95% CI (1.45, 10.15) times being infested by *pediculosis capitis* as compared to those having good knowledge, attitude and practice respectively (Table 3).

**Table 3:** Associated factors of pediculosis capitis in regression of bivariate and multivariate analysis among school-aged children in Woreta town, 2018 (n = 402)

Variables	Categories	Pediculosis		COR (95% CI)	AOR (95% CI)
		Yes	No		
Sex	Male	102	84	1	1
	Female	162	54	2.47 (1.62, 3.77)	3.29 (1.94–5.59)***
Age in years	5 to 8	60	25	1.77 (0.94,0.34)	2.19 (0.96, 5.01)
	9 to 11	154	76	1.49 (0.90, 2.49)	2.04 (1.07, 3.87)^*^
	≥ 12	50	37	1	*1*
Mothers education	Illiterate	136	35	5.05 (2.76, 9.24)	3.57 (1.74, 7.33)***
	Elementary	98	64	1.99 (1.13, 3.52)	1.68 (0.86*–*3.28)
	Secondary and above	30	39	1	1
Sharing hair comb	Share	153	34	4.2 (2.67, 6.67)	2.72 (1.58, 4.69)***
	No share	111	104	1	1.00
Knowledge towards *pediculosis capitis*	Poor	144	22	6.33 (3.78, 10.60)	2.51 (1.24, 5.10)*
	Good	120	116	1	1
Attitude towards *pediculosis capitis*	Poor	183	35	6.65 (4.18, 10.56)	2.42 (1.28, 4.60)**
	Good	81	103	1	1
Practice towards *pediculosis capitis*	Poor	80	6	9.56 (4.05, 22.59)	3.84 (1.45, 10.15)**
	Good	184	132	1	1

*COR* crude odds ratio, *AOR* adjusted odds ratio, *CI* confidence interval

Significant at * p ≤ 0.05, ** p ≤ 0.01, *** p ≤ 0.001, Hosmer–Lemeshow goodness-of-fit (p = 0.436)

### Discussion

Lice infestation is a major public health problem to which primary school students are more prone across the globe particularly in developing states. In this study, prevalence of *pediculosis capitis* was 65.7%. A study conducted in Southeast Iran reported similar findings with prevalence of 67.3% [10]. The prevalence of the current study is higher than a report from Bangkok, 23.32% [11], Iran 10.5% [17], Bilbao 9.39% [12], central Iran 29.35% [18], Argentina; 42.7% [19] and Cambodia 15.1% [20]. This difference might be related to differences in sample size, geo-cultural and socioeconomic variations. The prevalence in the current study is lower than a study conducted in Ratchaburi reporting 86.5% prevalence [21]. This difference might be due to sample size and socioeconomic factors.

In the current study sex, age, maternal education, sharing hair comb, knowledge, attitude and practice were significantly associated with *pediculosis capitis* infestation.

The odds of being infested by *pediculosis capitis* was 3.29 times higher in female students than males. This disparity is in line with previous studies from Bangkok [11], Iran [17, 22] Argentina [19] and Colombia [23]. This might be due to the habit of female students to have long hairs that can harbor the parasite and have close relationships with other girls, involving multiple and intimate body contact than boys [24]. Where as in another study in Iran the prevalence of *pediculosis capitis* infestation was not associated with sex of children [25].

Children age level was one of the predictors of *pediculosis capitis* infestation in the current study. Children aged 9 to 11 years were twice at higher risk of being infested by *pediculosis capitis* than those aged 12 years and above. This might be due to a higher level of personal hygiene practice among children with greater age. This result is supported by previous study done among primary schoolchildren in Kurdistan province [8].

Mothers’ education level was one of the predictors of *pediculosis capitis* infestation among schoolchildren. Children with illiterate mothers were 3.57 times at higher risk of being infested than children whose mothers’ educational status is secondary and above. This is in agreement with other studies [10, 17]. This might be because education is correlated with eagerness and ability to gain new knowledge and knowledge may help to have good personal hygiene practice which reduces infestation. However, in other studies maternal education was not associated [22, 26].

Students who shared comb were 2.7 times more likely to be infested than those who do not share comb. Sharing articles such as comb was significantly associated with pediculosis infestation in previous studies, too [10, 27, 28]. This is due to the fact that sharing comb is an efficient mechanism of louse and egg transmission. In contrast to this in an epidemiological study conducted in Asadabad, Iran sharing comb was not significantly associated with head lice infestation [26].

The knowledge of students regarding head lice infestation was significantly associated with infestation status. This is in line with a previous study [29]. This may be due to the fact that knowledge deficiency leads to inadequate ability to manage lice infestation [30]. The attitude towards *pediculosis capitis* and hygiene practice of schoolchildren were also significantly associated with *pediculosis capitis* infestation in the current study.

### Conclusion

Head lice infestation is a major public health problem and needs educational campaigns targeting mothers and planning of knowledge, attitude and practice improvement strategies by national and regional health authorities.

## Limitations of the study

The limitation of the current study were the inherent weakness of cross-sectional study design to establish cause-effect relationship, recall and social desirability bias.

## Data Availability

The dataset in the current study is available from the corresponding author upon request.

## Abbreviations

AOR: adjusted odds ratio
COR: crude odds ratio
CI: confidence interval
EPI Info: epidemiological information
SPSS: statistical package for social sciences
